# Environmental Health and Toxicology: Immunomodulation Promoted by Endocrine-Disrupting Chemical Tributyltin

**DOI:** 10.3390/toxics11080696

**Published:** 2023-08-12

**Authors:** Ricardo Correia da Silva, Mariana Pires Teixeira, Luciana Souza de Paiva, Leandro Miranda-Alves

**Affiliations:** 1 Laboratório de Endocrinologia Experimental-LEEx, Instituto de Ciências Biomédicas, Universidade Federal do Rio de Janeiro, Rio de Janeiro 21941-902, Brazil; rcorreia.ufrj@gmail.com (R.C.d.S.); mari.piress@gmail.com (M.P.T.); 2 Programa de Pós-Graduação em Ciências Morfológicas, Instituto de Ciências Biomédicas, Universidade Federal do Rio de Janeiro, Rio de Janeiro 21941-902, Brazil; 3 Programa de Pós-Graduação em Endocrinologia, Faculdade de Medicina, Universidade Federal do Rio de Janeiro, Rio de Janeiro 21941-902, Brazil; 4 Departamento de Imunobiologia, Instituto de Biologia, Universidade Federal Fluminense, Niterói 24210-201, Brazil; 5 Programa de Pós-Graduação em Patologia, Faculdade de Medicina, Universidade Federal Fluminense, Niterói 24210-201, Brazil; 6 Programa de Pós-Graduação em Farmacologia e Química Medicinal, Instituto de Ciências Biomédicas, Universidade Federal do Rio de Janeiro, Rio de Janeiro 21941-902, Brazil

**Keywords:** endocrine-disrupting chemical, tributyltin, immune system, organotin, cytokines, immunotoxicity

## Abstract

Tributyltin (TBT) is an environmental contaminant present on all continents, including Antarctica, with a potent biocidal action. Its use began to be intensified during the 1960s. It was effectively banned in 2003 but remains in the environment to this day due to several factors that increase its half-life and its misuse despite the bans. In addition to the endocrine-disrupting effect of TBT, which may lead to imposex induction in some invertebrate species, there are several studies that demonstrate that TBT also has an immunotoxic effect. The immunotoxic effects that have been observed experimentally in vertebrates using in vitro and in vivo models involve different mechanisms; mainly, there are alterations in the expression and/or secretion of cytokines. In this review, we summarize and update the literature on the impacts of TBT on the immune system, and we discuss issues that still need to be explored to fill the knowledge gaps regarding the impact of this endocrine-disrupting chemical on immune system homeostasis.

## 1. Introduction

### 1.1. Tributyltin

Organotin compounds are tin-based chemicals made up of hydrocarbons [1]. The use of these compounds ranges from industrial to agricultural biocidal agents such as antifungals, acaricides and molluscicides, and they are applied as wood preservatives and used in antifouling paints. Tributyltin (TBT), one of these organotin compounds, is a general name used to refer to a set of compounds distinguished by the presence of the (C4H9)3Sn group and low water solubility, with tributyltin oxide being a famous example [2,3]. TBT began to be widely used in antifouling paints in the mid-1960s, given its low cost and effectiveness in protecting ship hulls and underwater marine equipment against biofouling (TBT hinders the growth of algae, barnacles, mollusks and other organisms on ships’ hulls) [4].

After years of extensive use, adverse effects of TBT were described as it spread into the marine environment, where it is harmful to aquatic organisms [5]. For example, in the invertebrate *Nucella lapillus*, a low concentration of 1 ng/L was able to induce imposex, the development of male sexual characteristics in females, as seen in populations of this invertebrate along the coast of the United Kingdom [6]. In the commercial oyster *Crassostrea gigas*, a concentration of 20 ng/L of TBT affected larval growth, and concentrations smaller than 2 ng/L inhibited its calcification, making it impossible to fish for oysters in Arcachon Bay, France [7]. The imposex effect has also been described in vertebrates. The first report of imposex in vertebrates showed that doses of 0.1 µg/g of TBT present in the diet of *Paralichthys olivaceus* were sufficient for the masculinization of females [8]. Up to 2011, the imposex effect of TBT has been described in more than 260 species of marine gastropods. Moreover, by this time, environmental damage and economic losses had already spread over several continents [9].

Several restrictions against TBT use in antifouling paints were first imposed in France, the United Kingdom and other developed countries during the 1980s [10]. Years later, the International Maritime Organization (IMO) passed a global ban on the application of antifouling paints containing TBT (from 1 January 2003) and a ban on their presence on the surface of ships’ hulls (from January 2008) [11]. Even so, in 2004, it was estimated that 70–80% of the world naval fleet used TBT copolymer in its composition, given the economic benefits [10]. Also, in 2014, a United States company was fined for clandestinely producing and selling antifouling paints with TBT to various regions of the Caribbean. Nonetheless, the problem persists because, as recently as 2021, these paints could still be found for sale on the internet and be sent to different regions such as the Caribbean, Central America and Oceania [12].

Several factors can increase the half-life of TBT in the environment, such as its ability to be deposited in marine sediments (because TBT compounds exhibit significant lipid solubility and are preferentially absorbed by organic matter in soils or sediment) and to continue to be released into the environment for up to 100 years, according to mathematical models [4,13]. These factors have contributed to TBT values exceeding 7000 ng Sn/g in an environmental reserve in the Virgin Islands [14]. TBT also bioaccumulates in several marine species that are at the base of the food chain [15]. TBT can be found in these organisms even after 20 years of the initial contamination, maintained by their lipid solubility. Furthermore, the biomagnification of contamination occurs in the food chain, making it possible to find TBT residues in fish, seabirds and marine mammals [16].

Given the risk to human health due to the ingestion of products contaminated with TBT (e.g., seafood, water), the World Health Organization (WHO) has defined an acceptable daily intake value of 250 ng/Kg/day of TBT [17]. This value was extrapolated by a factor of 100 due to toxicity, kinetics and inter-individual differences tests performed in rats, in which the addition of TBT to the diet led to a reduction in the weight and function of the thymus of these animals [18]. Human exposure to TBT mainly occurs through the consumption of contaminated water and beverages. However, the consumption of marine food, in particular, has also been identified as a significant pathway for human exposure [19,20,21]. In Taiwan, TBT concentrations in oysters ranged from 320 to 1510 ng g^−1^ dry wt., depending on sampling locations. The highest TBT concentration (86–91% of total butyltin compounds) was 1510 ng g^−1^ dry wt., found in oysters from the Hsiangshan coastal area. Fishermen showed oyster consumption values of 94.1 and 250 g d^−1^ for typical and maximum exposure, respectively. The maximum intake of 250 g d^−1^ by fishermen was almost twice that of the general population (139 g d^−1^), indicating potential health risks for those exposed to these contaminated oysters [21]. In Portugal, 32% of the 28 duplicate diet samples from members of the University of Aveiro showed the presence of organotin compounds. These compounds were detected at relatively low levels, with TBT being found in only two of the samples [22]. In “Isla Grande Atacama,” northern Chile, the daily ingestion of 90 to 173 g of *Thaisella chocolata* (equivalent to four to eight organisms) from the most contaminated sites resulted in the consumption of TBT levels that exceeded the tolerable daily intake recommended by the European Food Safety Authority for tin (not exceeding 0.0015% in food composition and 100 ng Sn Kg^−1^) [23]. Nevertheless, it is important to highlight that distinct diets may result in different levels of TBT contamination in human blood and tissues, but this is not the only determining factor for exposure to TBT [5].

Studies have shown that chronic exposure to TBT, even in values lower than the acceptable daily intake, may lead to different complications. Newborn C57BL/6J offspring exposed in utero to 0.05 or 0.5 mg/Kg of TBT (administered to mothers via intraperitoneal injection every 24 h from the 12th day of gestation) exhibited accelerated adipocyte differentiation in the liver, testis and mammary glands. In adulthood, these offspring displayed increased epididymal adipose mass [24]. Moreover, exposure of stem cells derived from 8-week-old C57BL/6J mice, which were previously exposed to TBT (0.1 mg/Kg) in utero starting at the 16th day of gestation, to 50 nM of TBT for 14 days resulted in the predominant induction of adipogenesis over osteogenesis. These findings suggest that prenatal TBT exposure alters the differentiation potential of stem cells, favoring adipogenic lineage commitment [25]. Female rats treated with 100 ng/Kg/day of TBT showed signs of dysfunction of the hypothalamic–pituitary–adrenal axis, including inflammation, oxidative stress and fibrosis [26]. Additionally, treatment of female rats with 100 or 500 ng/Kg/day of TBT via gavage resulted in abnormalities in renal function, including decreased glomerular filtration rate, elevated levels of proteinuria, inflammation, oxidative stress and kidney fibrosis [27]. Furthermore, an extensive amount of toxic effects due to TBT have already been described, mainly related to endocrine, metabolic and reproductive dysfunctions [28,29,30,31,32,33].

Finally, another important adverse effect of TBT is its immunotoxicity. In the present review, we aim to summarize and update the current literature on the impact of TBT on the immune system, opening the door for new questions about the impact of this endocrine disruptor.

### 1.2. The Immune System

In response to a stimulus, like a challenge by a pathogen, the immune system generates innate and adaptive immune responses. Briefly, the innate response is immediate, less specific and primarily involves the action of monocytes, neutrophils and natural killer (NK) cells. In contrast, the adaptive response is built gradually, being more specific and long-lasting. It is mainly orchestrated by B lymphocytes, the production of antibodies and by T lymphocytes that coordinate cell-mediated immune response [34,35].

Regarding T lymphocytes, the population of Tαβ lymphocytes is mainly divided into two main subpopulations, CD4+ and CD8+ T lymphocytes, named according to their expression of some surface protein, called clusters of differentiation (CD). These cells have different functions. CD8+ T lymphocytes can induce the death of tumor cells or cells infected by viral pathogens via class I major histocompatibility complex (MHC) recognition and produce inflammatory mediators (interleukin (IL)-2, interferon gamma (IFN-γ) and tumor necrosis factor alpha -TNF-α) [36,37]. CD4+ T lymphocytes are characterized by the recognition of class II MHC, expressed on the surface of antigen-presenting cells (APCs), and can differentiate into different subpopulations depending on the cytokines present in the microenvironment [36,38]. The main subpopulations of CD4+ T lymphocytes include: T helper (Th)1 cells, which are capable of promoting a cell-mediated effector response against viruses, intracellular bacteria and protozoa, and are characterized by the production of cytokines such as IFN-γ and IL-2 [39]; Th2 cells, which are involved in the coordination of the humoral response, mainly against parasites (for example, helminths), as well as in the pathophysiology of several allergies (including asthma and atopic dermatitis), and are characterized by the production of IL-4, IL-5 and IL-13 [36,39]; and Th17 cells, which are involved in the defense against pathogens in the mucosa and in autoimmune diseases, where there is a hyperactivation of Th17 (as in rheumatoid arthritis), and are characterized by the production of IL-17 and IL-22 [36,40,41]. Regulatory T cells are another important subtype of T lymphocytes. These have a primary function of suppressing the activation of the immune system, more specifically inhibiting the activation and expansion of CD4^+^ and CD8+ T lymphocytes and B cell activation, preventing the exacerbation of inflammation. Regulatory T cells can be characterized by the expression of CD4, CD25 and FOXP3 molecules and by the secretion of granzyme B, TGF-β and IL-10 [42].

The balance of synthesis and secretion (autocrine and paracrine signaling) of different cytokines by immune cells and other cell types, such as endothelial cells, fibroblasts and bone marrow cells, maintains the multicellular network of communication in the microenvironment [43]. In fact, cytokines are the basis of communication for the initiation, maintenance and termination of immune responses to infections, and any change in them can lead to extremes, such as immunosuppression or the establishment of chronic inflammation [44]. TNF-α, for example, modulates the function of effector immune cells, such as neutrophils, promoting an increase in their activity, adherence and chemotaxis. On the other hand, IFN-γ promotes an increase in the antitumor activity of natural killer cells, the phagocytic activity of macrophages and the production of nitric oxide, in addition to the increased expression of MHC class I and II molecules. Interleukin-1β (IL-1β) affects the maturation, proliferation and synthesis of immunoglobulins by B lymphocytes, in addition to stimulating the synthesis of acute-phase proteins at the beginning of the inflammatory process [43,45,46]. In contrast, other cytokines, such as IL-10, have an immunoregulatory function, inhibiting the synthesis of cytokines such as IFN-γ, TNF-α, IL-12, IL-2 and IL-1β, thus inhibiting the inflammatory process and promoting a change in the profile of macrophages from the activated state to the tissue-resident phenotype [43,47,48].

**Figure 1:**
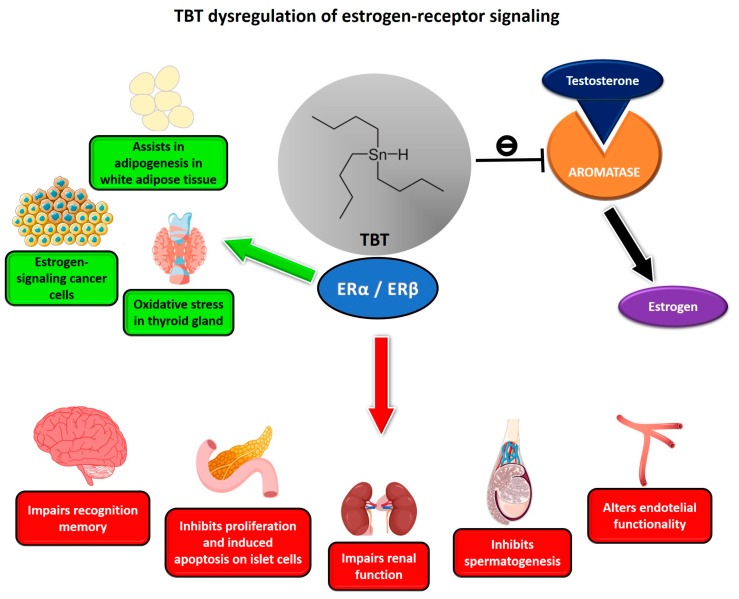
TBT impacts estrogen action and signaling. TBT may affect different systems by positively (green) or negatively (red) modulating ERα and ERβ, acting as an agonist or antagonist depending on the model. Additionally, TBT can block aromatase activity, preventing the conversion of testosterone to estrogen, leading to masculinization of female gastropods, for example.

It is worth noting that the immune system does not act alone; it functions in close connection with other systems, such as the endocrine system [49]. The mechanism of communication between the immune and endocrine systems occurs in “two-way” process, as some immune cells are capable of producing hormones and are also sensitive to hormonal action [50,51,52,53,54,55]. A good example is that of estrogen, which can modulate the production of cytokines, as well as the differentiation, proliferation and even apoptosis of cells of the immune system such as T and B lymphocytes, mast cells, basophils and eosinophils via estrogen receptors (ERs) that are expressed by these cells [54]. Cell populations of the immune system may differ in the expression of ERs, such as CD4+ T lymphocytes that express greater amounts of ERα than B lymphocytes, which, in contrast, express greater amounts of ERβ than CD4+ T cells. In parallel, CD8+ T lymphocytes express both ERα and ERβ in low amounts, but at equal rates [56,57]. Interestingly, TBT has also been described as an antagonist of human ERs by inhibiting the transcriptional activation of the ER-dependent reporter gene and the interaction between the ligand-binding domain of the β isoform (ERβ LBD) and the steroid receptor coactivator-1 (SRC1) [58,59]. Additionally, TBT acts as an inhibitor of aromatase, the enzyme accountable for the conversion of testosterone to estrogen, as well as the estrogen receptor in zebrafish, thereby reducing the effects of ethinylestradiol [60]. Additionally, numerous studies have revealed that TBT disrupts estrogen signaling, affecting various tissues, as shown in Figure 1 [28,30,33,61,62,63,64,65].

It is also known that different cytokines and chemokines have targets in the hypothalamic–pituitary–thyroid (HPT) axis. Deregulation in the balance of these molecules can impact the HPT axis, mainly thyroid function [66]. Therefore, exposure to endocrine disruptors like TBT can impact innate and/or adaptive responses, more specifically interfering with the cellular and humoral immune responses, as well as the lifespan of immune cells [67].

## 2. TBT and the Immune System

The relationship between TBT contamination and immunotoxicity was first observed in animals that live exclusively or mainly in aquatic environments. In in vivo experiments with the fish *Oncorhynchus mykiss*, TBT led to thymic atrophy and reduced circulating lymphocyte population [68]. Moreover, bottlenose dolphins (*Tursiops truncatus*) and sea otters (*Enhydra lutris*) that lived in contaminated sites were found dead along the US coast and had high tissue concentrations of TBT. The mortality of both species has been linked to a lower ability of the immune system to fight infectious diseases [69,70]. In vitro experiments using seal cells (*Phoca vitulina*) showed that doses of between 50 and 200 nM of TBT and its metabolite dibutyltin (DBT) reduced the antitumor capacity of NK cells and decreased the proliferation of T cells, and that 100–200 nM of DBT decreased macrophage phagocytic activity [71]. Over the last decades, tests have been carried out using animal models (mainly mice) and human cells, which will be addressed throughout this article, as a way of inferring whether TBT’s immunotoxic effects might also be seen in humans.

### 2.1. Mice Models

C57BL/6 mice that had TBT inserted in their diet for a period of two weeks showed a reduction in the number of lymphocytes in the spleen and lymph nodes, thymic atrophy and an increase in serum immunoglobulin M, but a decrease in immunoglobulin G [72]. In addition, BALB/c mice injected with 100 nM of TBT showed an increase in serum levels of the cytokines IFN-γ, TNF-α and IL-13 and the chemokines MIP-1β and RANTES. A decrease in the cytokine IL-2, which is essential for the maturation of B and T lymphocytes, was also observed [44]. In splenic cells of CBA/J mice stimulated in vitro with anti-mouse CD3 antibodies, exposure to 0.1 µM of TBT was sufficient to inhibit the secretion of IFN-γ and IL-4 by T CD4 cells [73]. ICR mice treated in vivo with doses of 4 and 20 mg/Kg of TBT showed a positive linear increase in thymocyte apoptosis and Fas expression, indicating that TBT may lead to cell apoptosis [74]. Furthermore, low doses of TBT (0.1, 1, 3 and 10 nM) were also capable of inducing apoptosis of Balb/c thymocytes in vitro via an increase in reactive oxygen species (ROS), a reduction in growth hormone (GH), depolarization of the mitochondrial membrane and the activation of caspase-3 [75]. In Balb/c and C57BL/6 T lymphocytes, it was seen that TBT (100 nM) in vitro induces the differentiation of T cells to the Th2 phenotype, which is characterized by the production of IL-10 and suppression of the production of IL-12, one of the main cytokines responsible for the differentiation of CD4+ T lymphocytes into the Th1 phenotype [76,77]. It was also observed that in C57BL/6 mice treated in vivo with TBT (6 μmol/Kg), there was greater ovalbumin-induced airway inflammation than in untreated mice. Furthermore, in the bronchoalveolar lavage fluid of these TBT-treated animals, there were increased numbers of eosinophils as well as increased IL-5 levels and IgE levels in serum, which was expected, given that Th2 cells are involved in the response to allergens [77]. Part of this effect was due to an increase in oxidative stress in secondary lymphoid organs of C57BL/6 animals promoted by TBT treatment, and, interestingly, Th17 lymphocytes had no role in the increased inflammation observed in the respiratory tract [78].

After oral treatment of ICR mice with 1, 10 and 20 mg/Kg of TBT, a decrease in the percentage of CD4+ and CD8+ T cell populations was observed in the thymus, but with an increase in the percentage of the CD4+CD8+ and CD4-CD8- T cell populations and decreased expression of IL-7 [79]. According to the same study, the spleens of ICR animals were also analyzed during TBT treatment, and a decrease in the percentage of naive CD44-CD62L+ and effector/memory CD44+ T cells was observed. Collectively, these data suggest that TBT impairs the development of T cells in the thymus and spleen [79]. Regarding B lymphocytes, in an C57BL/6 in vitro model, exposure to TBT promoted apoptosis of pro-B cells. In an C57BL/6 ex vivo model, TBT directly affected the differentiation of B cells, in addition to altering the bone marrow microenvironment [80]. In macrophages, exposure of the murine J774.1 cell line to TBT (1–1.5 µM) induced an increase in TNF-α expression and an increase in caspase-3 activity, leading to an increase in the apoptosis of these cells [81]. Additionally, treatment of the J774A.1 cell line with 0.4, 0.6, 1 and 1.2 µM of TBT promoted cell death possibly via receptor-interacting protein kinase 1 (RIP1) and receptor-interacting protein kinase 3 (RIP3), which are related to necroptosis. Furthermore, bone marrow-derived macrophages from TBT-treated C57BL/6 mice also displayed increased apoptosis [82]. Exposure to TBT also induced changes in RAW 264.7 murine macrophages, leading to activation of the inflammasome complex [83]. Finally, macrophages from C57BL/6 mice treated with doses of 250 and 500 µg/Kg of TBT showed activation of the peroxisome proliferator-activated receptor gamma (PPARγ) pathway and increased expression of genes related to lipogenesis and lipid metabolism, which could be related to a role of the innate immune system in the TBT-promoted obesogenic effect [84].

### 2.2. Human Models

In humans, TBT (1 μM) induced neutrophil apoptosis in vitro via a caspase-dependent mechanism [85]. Moreover, TBT (2.5–200 nM) in vitro decreased the viability of peripheral blood mononuclear cells (PBMCs) and was able to modulate the production of IL-1β and IFN-γ in a dose-dependent manner, with the TBT effect varying according to the concentration and duration of the exposure [86,87]. Also, in PBMCs and in the same concentrations of TBT (ranging from 2.5 to 200 nM) with varying in vitro exposures of 10 min, 1, 6 and 24 h, TBT induced the activation or increased expression levels of ribosomal protein S6 (S6), eukaryotic initiation factor 4B (eIF4B) and eIF4E in these cells. Surprisingly, this activation or elevation occurred at concentrations known to induce pro-inflammatory cytokine production, despite the absence of concomitant mRNA upregulation for these proteins [88]. Specifically, in monocyte-free human PBMCs, in addition to an increase in IL-1β synthesis and secretion, an increase in IL-6 synthesis and secretion was observed using the same concentrations of TBT [89,90]. These data were corroborated, given that the inhibition of Toll-like receptors (TLRs 4, 1/2 and 8) in PBMCs without monocytes treated with TBT leads to a significant decrease in the production of IL-1β and IL-6. This effect probably occurs because exposure to TBT causes the activation of these TLRs, promoting the activation of the MAPK pathway [91].

In human NKs, TBT also modulates IL-1β and TNF-α, promoting an increase in the secretion of these cytokines at low doses (5–50 nM for IL-1β and 5–100 nM for TNF-α), but a decrease at high doses (200 nM for both) [86,92]. Other in vitro studies also showed that NKs exposed to TBT (200–300 nM) had lower expression of perforins and granzyme B, as well as lower cytotoxic activity against tumors (TBT 25–500 nM) [93,94]. Additionally, within 10 min of TBT treatment, dosages ranging from 25 to 300 nM promoted the activation of MAP3K and its associated proteins, such as c-Raf and protein kinase C (PKC), in in vitro human NK cells. The activation of MAPK3 and apoptosis signal-regulating kinase 1 (ASK1) in human NK cells was another effect of TBT, but this occurred within an hour of exposure. In this approach, the innate potential of NK cells to effectively kill target cells may be disrupted by TBT, which could impair the activation of this pathway in a subsequent encounter with tumor cells or infected cells [95,96]. Specifically, in human B lymphocytes, in vitro treatment with TBT (100 nM) reduced the proliferation, survival and differentiation of mature B cells [97]. Finally, in human marrow cells, TBT (1 nM) led to a decrease in the percentage of CD19+CD22+ B cells in a mechanism independent of the PPARy pathway [98,99].

When exposed to concentrations of 0.2 and 0.5 μM of TBT for various times (3, 6, 12 and 24 h) and subjected to whole-genome gene expression microarray analysis, the human T lymphocyte cell line Jurkat revealed that TBT treatment elicits immunotoxic effects by inducing endoplasmic reticulum (ER) stress, subsequently leading to an increase in intracellular Ca^2+^ levels. This elevation in Ca^2+^ levels triggers the activation of the nuclear factor of activated T cells (NFAT) and nuclear factor-kappa B (NF-κB), resulting in T cell activation, the induction of oxidative stress and, ultimately, cell apoptosis [100]. In Jurkat cells, doses ranging from 200 nM to 1 μM of TBT also induced apoptotic responses within 1 to 24 h of treatment, with the recruitment of caspase-8 and caspase-10 by TRAIL-R2. Interestingly, in Jurkat cells deficient in caspase-8, the apoptotic effects of TBT are only slightly reduced, whereas the inhibition of caspase-10 prevents all TBT-induced apoptotic effects [101]. Additionally, in Jurkat cells knockout for DNA fragmentation factor 40 (DFF40), exposure to TBT for 24 h at concentrations of 0.2, 0.4 and 0.6 μM did not induce DNA fragmentation, apoptosis and ROS production at the same rate as observed in wild-type cells. These findings indicate that DFF40 may play an important role in regulating cellular susceptibility to TBT and its contribution to the maintenance of DNA stability [102].

### 2.3. Other Models

In a zebrafish model (*Danio rerio*), chronic exposure for 8 weeks with different doses of TBT (1, 10 and 100 ng/L) led to a decrease in the activity of antioxidant enzymes (superoxide dismutase (SOD), catalase and glutathione peroxidase), intestinal lysozyme and immunoglobulin M (IgM) and to an increased expression of TNF-α, IL-1β, IL-6, NF-κB p65 and heat shock proteins HSP70 and HSP90 in the intestines, indicating that TBT induces oxidative stress and immunotoxicity in zebrafish [103]. In another protocol of chronic exposure to TBT with zebrafish for 6 weeks at doses of 10, 100 and 300 ng/L, a decrease in the amount of lysozyme and IgM was also observed, along with a dysregulation in the production of thyroid hormones [104].

Unsaturated fatty acid levels in muscle tissue were increased in *Gobiocypris rarus* fish after chronic exposure to environmentally relevant amounts of TBT (1, 10 and 100 ng/L) for 60 days. Pro-inflammatory cytokines TNF-a, IL-1 and the NF-κB transcription factor were also upregulated in the muscle tissue, as was antioxidant enzyme activity, suggesting potential TBT effects on the growth of fish and their nutritional value [105]. Additionally, after being exposed to TBT concentrations of 50 and 500 ng/L for 60 days, the lined seahorse (*Hippocampus erectus*) showed significant tin accumulation, liver damage, changes in antioxidant defenses (including increased SOD activity and decreased catalase activity) and upregulation of 20 genes linked to antioxidant defense, immune responses and inflammation [106].

In *Takifugu obscurus* fish, different water concentrations of TBT (1.962, 3.924 and 9.81 μg/L) for 96 h led to increased production of ROS and concomitant upregulation of CD28 (a known costimulatory receptor present in the surface of T cells) in the gills and liver of these animals, suggesting that CD28 plays a role in the response to TBT toxicity [107]. Also, in the *Takifugu obscurus* model, the same concentrations of TBT in water (1.962, 3.924 and 9.81 µg/L) for 96 h and chronic exposure with 900 ng/L for 30 days induced lower mRNA expression of TLRs 2 and 3 in the gills and higher mRNA expression of TLR18 and TLR22 in the liver and gills when compared to animals not exposed to TBT, confirming that these tissues are vital sites in the initial response to TBT exposure [108].

### 2.4. TBT and Other Endocrine-Disrupting Chemicals

Sensitization to allergens is one of the side effects caused by endocrine disruptors in the immune system, as they induce the breakdown of homeostasis through changes in the production of cytokines and chemokines, as summarized in this work in relation to exposure to TBT (Table 1). In the context of bisphenol S (BPS), water intake of 0.4 μg/Kg/day of BPS for 6 weeks concomitant with exposure to ovalbumin (OVA) every 2 weeks increased lung inflammation in C3H/HeJ mice, as well as anti-OVA IgE and IgG1 levels in serum and IL-5, IL-13, IL-33 and eotaxin levels in bronchoalveolar lavage fluid [109]. In the mediastinal lymph nodes of these same animals, there was an increase in the number of total cells and antigen-presenting cells such as dendritic cells. In addition, a restimulation of lymph node cells with OVA in vitro led to an increase in cell proliferation and cytokine production of the Th2 lymphocyte profile, such as IL-4, IL-5 and IL-13, indicating that BPS may lead to an increase in the number of Th2 cells and greater sensitization to allergens. Similar effects have already been observed for TBT in C57BL/6 mice models [76,77,109]. Furthermore, increased levels of IL-4 and decreased levels of IL-12 were observed in the umbilical cord blood of newborns whose mothers had higher amounts of monoethyl phthalate (MEP), a metabolite of di-ethyl phthalate (DEP), in blood and urine (during weeks 24 to 28 of gestation), indicating a possible polarization of newborn T cells towards the Th2 phenotype [110]. Bisphenol A (BPA) at doses of 10, 30 and 50 µM for 12 h led to a greater translocation of the transcription factor NF-κB p65 and to an increase in the production of cytokines IL-1β, IL-6 and TNF-α, together with nitric oxide and prostaglandin E2 (PGE2), in murine macrophages of the RAW264.7 lineage, suggesting that BPA can induce a pro-inflammatory response in these cells [111].

In the context of cancer, a single dose of BPA (250 ug/Kg) in newborn Balb/c mice is capable of inducing lung metastasis with increased intratumoral production of IL-1β, IL-6, IFN-γ, TNF-α and VEGF in a model of induced mammary tumorigenesis in which animals were injected in situ with 4T1 tumor cells when they reach sexual maturity [112]. Interestingly, 10^−8^ M of BPA was also able to increase the migration of breast ductal carcinoma in situ (DCIS) cells and RAW264.7 macrophages in an in vitro co-culture system. In in vivo experiments with Balb/c mice, exposure to 2.5 µg/L of BPA for 70 days promoted an increase in DCIS primary tumor growth rate and lymph node metastasis and a concomitant increase in protumorigenic M2 macrophages [113]. It has already been observed that treatment of C57BL/6 mice with 4 mg/Kg of di(2- ethylhexyl) phthalate (DEHP) for 21 days before the injection of B16F10 melanoma cells and for 7 days after the injection, reduced the polarization of macrophages into the M1 profile, but increased the polarization into the M2 profile, leading to tumor formation and growth [114]. This same polarization for the M2 profile is seen during in vitro exposure to benzophenone-3 (BP-3) in primary human macrophages [115]. Currently, there are no published data on how the exposure of macrophages to TBT would alter the immune response of these cells in the context of cancer, as previously described for other endocrine disruptors.

Similar to TBT, individual and co-exposure to the disruptors mancozeb (8000 mg/Kg/day) and fipronil (95 mg/Kg/day) for 29 days by oral gavage led to immunotoxicity in the spleen and thymus of Swiss albino mice (being more prominent in the treatment with both disruptors), as indicated by lower organ weight and cellularity, lower proliferation of splenocytes and thymocytes and higher rates of apoptosis and necrosis of these cells [116]. However, injection of Swiss mice with 50 μg/Kg of BPA for 6 weeks led to an increase in the number of lymphocytes and monocytes in the blood and an invasion of lymphocytes and eosinophils into the red pulp of the spleen [117]. In addition, doses of 100 µM of BPA, BPS, BPF and dimethyl terephthalate (DMTP) led to lower proliferation and viability of B lymphocytes isolated from the spleen of mice and stimulated with LPS, with BPA being the most toxic for B cells among these [118]. Interestingly, even though TBT is a PPARy agonist, it can induce a reduction in the mature population of B lymphocytes regardless of activation of the PPARy pathway by inducing changes in the bone marrow microenvironment that lead to adipogenesis in favor of lymphopoiesis [59,80,98,99]. It is important to mention that other endocrine disruptors that are agonists of the PPARy pathway (such as phthalate metabolites) are well known to affect the differentiation of B lymphocytes in the bone marrow by inducing the apoptosis of B lymphocyte precursors [119,120,121].

## 3. Current Gaps in Literature

Many gaps need to be filled in the current knowledge about the effects of TBT on the immune system. For example, its effect on regulatory T cells is unclear; these cells are crucial for the maintenance of immune homeostasis through the synthesis and secretion of cytokines such as IL-10, TGF-β and IL-35 [122,123]. To date, there is only one study available in Japanese demonstrating that in the presence of TBT, regulatory T lymphocytes are more likely to enter apoptosis than Th2 lymphocytes. [124]. Moreover, TBT’s impact on the functionality of γδ T cells is still unknown. These cells are widely present in peripheral tissues, where they promote tissue repair and immune surveillance in barrier tissues through the synthesis and secretion of various molecules such as IFN-γ, IL-17, IL-22, keratinocyte growth factor (KGF), insulin growth factor 1 (IGF1) and fibroblast growth factor 9 (FGF9) [125].

Unfortunately, there are also no studies on the effect of TBT on dendritic cells, even though these cells are very important for the effectiveness of the immune system, given that they bridge the gap between innate and adaptive immunity by capturing, processing and presenting antigens to T lymphocytes, mediating their polarization into effector cells [126]. In the case of other disruptors, it has already been seen that in vitro exposure to a mixture of BPA and BPF (10–50 μM) led to a decrease in the differentiation and maturation of human monocyte-derived dendritic cells, in addition to the loss of endocytic capacity and suppression of activation of NF-κB and ERK 1/2 pathways [127]. In human plasmacytoid dendritic cells exposed to DEHP, inhibition of the NF-κB and ERK pathways was also observed, along with lower expression of IFN-α and IFN-β. This led to changes in the cytokine secretion profile of CD4+ T cells activated by these dendritic cells, suppressing the production of IFN-γ, but increasing the production of IL-13 [128].

Another relevant point for this discussion is the lack of data about the effects of TBT on the polarization of macrophages into M1 and M2 profiles, as already described for other endocrine disruptors. This knowledge is important because M1 macrophages are mainly involved in the inflammatory response with the secretion of cytokines such as IL-1, IL-6, IFN-γ and TNF-α, and M2 macrophages are mostly involved in anti-inflammatory responses, producing cytokines such as IL-10 and TGF-β, so an imbalance between the two populations leads to the fatal loss of immune homeostasis [129].

There are only two studies on mast cells involving TBT. The first, from 30 years ago, shows that in vitro exposure of rat serosal mast cells (2.4 × 10^5^ in 0.8 mL of medium) to 1 mM of TBT for 5 min leads to a strong inhibition of histamine secretion [130]. The second, a study of the impact of TBT on the coronary function, only reports an increase in mast cells in cardiac vessels of Wistar rats exposed to 100 ng/Kg of TBT per day by oral gavage [131]. As mast cells are cells present in all (but not only) mucosa of the body and synthesize and secrete various products such as IL-4, IL-6, TGF-β, biogenic amines, growth factors and proteases [132], the lack of knowledge regarding TBT’s effect these cells is worrisome, since these cells may contribute to inflammation in the respiratory system. In fact, there are already enough data to demonstrate that exposure to TBT causes inflammation in the respiratory system [76,77,78].

Moreover, to the date of this publication, there are also no data comparing immunological alterations in males and females exposed to TBT. Evidence for divergence in responses between both sexes has already been shown for other endocrine disruptors, such as bisphenols [133] and even for TBT, but this was in the context of the nervous system [134].

## 4. Conclusions

**Figure 2:**
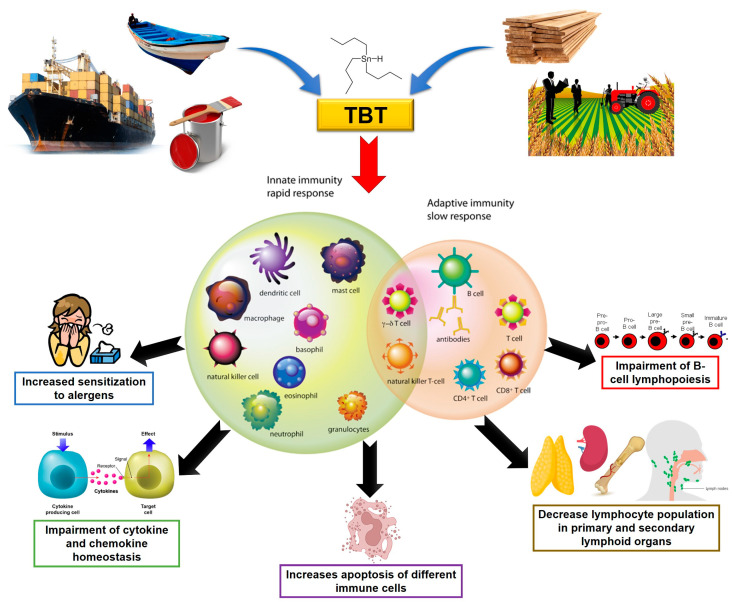
Summary of the main effects of TBT on the immune system.

In this article, we summarize the existing knowledge about the effects of TBT on immune system homeostasis. Studies using different animal and human models have shown that exposure to TBT is able to alter the function and viability of immune cells, which may impact immune responses. Changes in the homeostasis of cytokine and chemokine production (summarized in Table 1) were also described in human PBMCs and NK cells. Furthermore, murine models have shown similar alterations in cytokine profiles in the serum of TBT-exposed animals, as well as thymic atrophy and changes in cell populations of primary and secondary lymphoid organs. These changes, along with others directly or indirectly induced by TBT, lead to an increased sensitization of mice to allergens, as seen by the increase in the differentiation of CD4+ T lymphocytes to the Th2 profile, the decrease in the lymphocyte population present in secondary lymphoid organs and the apoptosis of murine thymocytes. Finally, in both human and murine models, TBT directly impacts B cell lymphopoiesis, decreasing the mature B cells (Figure 2).

Here, we have reviewed decades of observation and experimentation studies on the effects of TBT on the immune system. We hope that this work not only brings light to that which is already known about TBT, but that it also highlights the scarcity of our knowledge about this topic and the urgent need for additional studies. Moreover, we hope to call attention to the impacts of TBT on the environment and on human health, even in doses considered safe by government agencies worldwide.

## Figures and Tables

**Table 1:** Summary of changes in cytokine/chemokine synthesis and/or secretion caused by TBT treatment.

Cytokine/Chemokine	Experimental Model	Effect of TBT
IL-1β	Human/in vitro—zebrafish—*Gobiocypris rarus*	- Increased synthesis and secretion at low dose, inhibited at high dose [86,87,89]. - Increased expression in the intestine [103]. - Increased expression in muscle [105].
IL-2	BALB/c	- Decreased in serum [44].
IL-4	C57BL/6 and CBA/J mice spleen cells	- Indirect increase [78]. - Inhibited in vitro [73].
IL-5	C57BL/6	- Increased in serum [77].
IL-6	Human/in vitro—zebrafish	- Increased synthesis and secretion [89,90]. - Increased expression in the intestine [103].
IL-7	ICR mice	- Decreased expression in the thymus [79].
IL-10	C57BL/6	- Indirect increase [76,77].
IL-12	C57BL/6	- Indirect decrease [76,77].
IL-13	BALB/c	- Increased in the serum [44].
IFN-γ	BALB/c, CBA/J mice spleen cells and human/in vitro	- Increased in serum [44]. - Increased synthesis and secretion at low dose, inhibition at high dose [86,87]. - Inhibited in vitro [73].
TNF-α	BALB/c/J774.1 cell line and human/in vitro—zebrafish and *Gobiocypris rarus*	- Increased in serum [44]. - Increased synthesis [81]. - Increased synthesis and secretion at low dose, inhibition at high dose [86,92]. - Increased expression in the intestine [103]. - Increased expression in muscle [105].
MIP-1β	BALB/c	- Increased in serum [44].
RANTES	BALB/c	- Increased in serum [44].

## Data Availability

The images in this study were generated using the PowerPoint software and created from freely available digital image repositories (pngtree.com; pngwing.com accessed on 4 August 2023).
